# Genome-Wide Association Study of Leaf Rust and Stem Rust Seedling and Adult Resistances in Tetraploid Wheat Accessions Harvested in Kazakhstan

**DOI:** 10.3390/plants11151904

**Published:** 2022-07-22

**Authors:** Yuliya Genievskaya, Nicola Pecchioni, Giovanni Laidò, Shynar Anuarbek, Aralbek Rsaliyev, Vladimir Chudinov, Alibek Zatybekov, Yerlan Turuspekov, Saule Abugalieva

**Affiliations:** 1 Laboratory of Molecular Genetics, Institute of Plant Biology and Biotechnology, Almaty 050040, Kazakhstan; julia.genievskaya@gmail.com (Y.G.); shinar_anuar92@mail.ru (S.A.); alexbek89@mail.ru (A.Z.); yerlant@yahoo.com (Y.T.); 2 Faculty of Biology and Biotechnology, Al-Farabi Kazakh National University, Almaty 050040, Kazakhstan; 3 Research Centre for Cereal and Industrial Crops, 71122 Foggia, Italy; nicola.pecchioni@crea.gov.it (N.P.); giovanni.laido@crea.gov.it (G.L.); 4 Laboratory of Phytosanitary Safety, Research Institute of Biological Safety Problems, Gvardeisky 080409, Kazakhstan; aralbek@mail.ru; 5 Breeding Department, Karabalyk Agricultural Experimental Station, Nauchnoe 110908, Kazakhstan; ch.den@mail.ru

**Keywords:** tetraploid wheat, association mapping, leaf rust, stem rust, quantitative trait loci, single-nucleotide polymorphism

## Abstract

Leaf rust (LR) and stem rust (SR) are diseases increasingly impacting wheat production worldwide. Fungal pathogens producing rust diseases in wheat may cause yield losses of up to 50–60%. One of the most effective methods for preventing such losses is the development of resistant cultivars with high yield potential. This goal can be achieved through complex breeding studies, including the identification of key genetic factors controlling rust disease resistance. The objective of this study was to identify sources of tetraploid wheat resistance to LR and SR races, both at the seedling growth stage in the greenhouse and at the adult plant stage in field experiments, under the conditions of the North Kazakhstan region. A panel consisting of 193 tetraploid wheat accessions was used in a genome-wide association study (GWAS) for the identification of quantitative trait loci (QTLs) associated with LR and SR resistance, using 16,425 polymorphic single-nucleotide polymorphism (SNP) markers in the seedling and adult stages of plant development. The investigated panel consisted of seven tetraploid subspecies (*Triticum turgidum* ssp. *durum*, ssp. *turanicum*, ssp. *turgidum*, ssp. *polonicum*, ssp. *carthlicum*, ssp. *dicoccum*, and ssp. *dicoccoides*). The GWAS, based on the phenotypic evaluation of the tetraploid collection’s reaction to the two rust species at the seedling (in the greenhouse) and adult (in the field) stages, revealed 38 QTLs (*p* < 0.001), comprising 17 for LR resistance and 21 for SR resistance. Ten QTLs were associated with the reaction to LR at the seedling stage, while six QTLs were at the adult plant stage and one QTL was at both the seedling and adult stages. Eleven QTLs were associated with SR response at the seedling stage, while nine QTLs were at the adult plant stage and one QTL was at both the seedling and adult stages. A comparison of these results with previous LR and SR studies indicated that 11 of the 38 QTLs are presumably novel loci. The QTLs identified in this work can potentially be used for marker-assisted selection of tetraploid and hexaploid wheat for the breeding of new LR- and SR-resistant cultivars.

## 1. Introduction

Tetraploid wheat (*Triticum turgidum* L.) is an important species within the genus *Triticum*, which harbors many desirable agronomic traits [1,2]. Durum wheat (*Triticum turgidum* L. subsp. *durum* [Desf.] Husn.) is the primary wheat used for pasta and semolina production, which has high economic importance. Kazakhstan produces 472,000 t of durum wheat and exports 385,000 t of grain annually [3].

Like other cereal crops, tetraploid wheat is subject to many serious infections, including fungal diseases. The most significant ones are called rusts and are caused by fungi of the genus *Puccinia* Pers. Rust diseases are increasingly becoming the largest threat to wheat production [4]. Among the various wheat rust diseases, leaf rust (LR) caused by *Puccinia triticina* Eriks. (*Pt*) and stem rust (SR) caused by *Puccinia graminis* f.sp. *tritici* (*Pgt*) are the most common in many wheat-growing areas around the world and may cause substantial yield losses [5]. *Pt* and *Pgt* are able to infect both durum wheat and hexaploid bread wheat (*Triticum aestivum* L.). Among all wheat diseases, SR is historically the most damaging disease worldwide [6,7] as, under suitable conditions, grain yield losses of 70% or even more are possible. SR has caused serious yield losses of spring wheat in Kazakhstan, particularly in northern Kazakhstan, where spring wheat production prevails; for example, in the 2015 growing season in the Kostanai region, North Kazakhstan, and the adjacent Omsk region of Russia, the stem rust epidemic covered more than 1 million hectares [8]. In the period 2017–2018, SR occurred again, involving not only the northern regions of Kazakhstan but also Eastern Kazakhstan, Omsk, Novosibirsk, and the Altai Krai regions of Russia [9,10]. Both of these outbreaks demonstrated 70–90% severity of SR infection and, as a result, there was a significant decrease not only in the yield but also in the quality of bread wheat grain [11]. Such SR epidemics have occurred periodically, not only in Kazakhstan and adjacent territories. In 2013, a local epidemic of wheat SR in Germany was followed by infections in Denmark, Sweden, and the U.K. [12]. Another large outbreak of SR was registered in Sicily in 2016, affecting thousands of hectares of both durum and bread wheat [13]. LR can also cause damaged wheat yields and, if the infection is severe and occurs before heading time, it may cause up to 30–40% yield loss [14]. In Kazakhstan, between 2001 and 2016, the mass spread of the pathogen—alone or in combination with Septoria blight—occurred eight times [15]. Commercial wheat cultivars in Kazakhstan have demonstrated poor resistance to LR and, in the case of early disease manifestation and strong development, LR may cover an area of up to 1.5–2.0 million hectares and reduce the yield by 15–20% [16,17]. As for the epidemics of LR worldwide, in 2001, a severe LR infection of durum wheat was reported in Mexico [18]. During the 2001–2002 season, LR epidemics of durum wheat were recorded at many locations in Spain, where durum wheat prevails [19]. As *Pt* and *Pgt* spores are easily dispersed by the wind over large distances, the pathogens constantly change their virulence, new highly virulent races may occur, and epidemic outbreaks are difficult to predict anywhere in the world. Traditionally, over the past 10 years, the main control of rust infections has been through the application of fungicides. Although this method is universal, it is destructive to ecosystems and may cause serious ecological problems. A good alternative to fungicides is the development and usage of cultivars with broad genetic resistance to *Pt* and *Pgt*.

The genetic background of wheat resistance to fungal diseases is complex due to the quantitative nature of the traits [20] and is additionally complicated by the variability of pathogen races in certain environments [21]. Approximately 60 *Sr* and nearly 80 *Lr* resistance genes have been identified in bread and durum wheat and their diploid relatives [22]. Among them are R genes, which are pathogen race-specific and effective at all plant growth stages, and adult plant resistance (or APR) genes, which are functional only in adult plants [20]. Both resistance gene types are important and should be accounted for in the development of cultivars with broad resistance to fungal diseases. In addition to the known *Lr* and *Sr* genes, there are hundreds of quantitative trait loci (QTLs) for rust resistance in the literature identified using linkage mapping [23,24,25,26,27] and association mapping [28,29,30,31,32] approaches. Association mapping, or genome-wide association study (GWAS), has become an increasingly preferable approach recently, as this method considers more genetically diverse panels. The main source of information for genetic polymorphisms is single-nucleotide polymorphism (SNP) markers. SNP markers are abundant in the wheat genome and can be easily detected using array-based genotyping platforms, such as the Illumina Wheat 9K iSelect SNP array [33], Illumina Wheat 90K iSelect SNP genotyping array [34], Wheat 15K SNP array [35], Axiom^®^ Wheat 660K SNP array, Wheat 55K SNP array, Axiom^®^ HD Wheat genotyping (820K) array [36], Wheat Breeders’ 35K Axiom array [37], or the Wheat 50K Triticum TraitBreed array [38]. Many of these have been used for mapping QTLs in durum wheat associated with yield components [39], grain quality [40], abiotic resistance [41], and disease resistance [42], including against LR [43] and SR [44].

In Kazakhstan, research on wheat disease resistance has been ongoing over the past 10 years [11,45,46,47,48]; however, studies focused on the assessment of durum wheat resistance have been limited. For instance, it has been shown that, at both the seedling and adult plant stages, the majority of durum wheat accessions from Kazakhstan were susceptible in fungal multi-pathogen tests, where only several accessions demonstrated moderate resistance [45]. Although no comprehensive GWASs for resistance to rust pathogens of tetraploid wheat have been attempted, a GWAS for yield-associated traits using a tetraploid wheat collection harvested in Kazakhstan has recently been reported [49], providing a platform for searching QTLs associated with LR and SR resistances. Therefore, the purpose of the current study was to identify QTLs for race-specific seedling resistance to common local *Pt* and *Pgt* races under greenhouse conditions, as well as APR to LR and SR in tetraploid wheat grown under natural conditions in northern Kazakhstan, which is the most common durum wheat-growing region in the country.

## 2. Results

### 2.1. Seedling Resistance to Pt and Pgt Races

**Figure 1:**
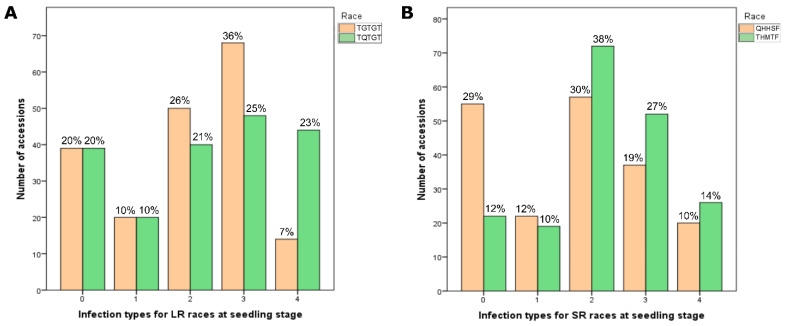
Summary of infection types among 193 tetraploid wheat cultivars and breeding lines infected with races of (**A**) *Puccinia triticina* Eriks. (LR) and (**B**) *Puccinia graminis* f. sp. *tritici* (SR) at the seedling stage.

The collection of tetraploid wheat was assessed for resistance to the two *Pt* and two *Pgt* races using two replicates and their mean values. The distribution of accessions with respect to their *Pt* and *Pgt* infection types at the seedling growth stage is presented in Figure 1. The largest part of the studied collection (36%) was moderately susceptible (3, MS) to the *Pt* race TGTGT, while the smallest part (7%) was susceptible (4, S) to the same race. The distribution by other infection types for the race TGTGT was as follows: 26% moderately resistant (2, MR), 20% immune (0, I), and 10% resistant (1, R). As for the second studied *Pt* race (TQTGT), the distribution of accessions among infection types was more even: 25% MS, 23% S, 21% MR, 10% R, and 20% I. Thus, the proportions that were resistant (0–2 or I, R, and MR infection types) and susceptible (3 and 4 or MS and S infection types) to the two *Pt* race accessions at the seedling stage were 53.5% and 46.5%, respectively.

The assessment of resistance to the *Pgt* race QHHSF showed the largest part of the collection (30%) to be MR, followed by a nearly equal part (29%) I, 19% MS, 12% R, and 10% S. For the second *Pgt* race, THMTF, the distribution of infection types was different: 38% MR, 27% MS, 14% S, 12% I, and 10% R. Thus, in the case of the two *Pgt* races, on average, the largest part of the collection (65.5%) was resistant, while the susceptible part was smaller (34.5%).

Eight accessions (5-BIL42, Athena, Cannizzo, Kronos, Orfeo, Tito, Tiziana, and PI 289606) in the studied tetraploid wheat collection demonstrated total immune reaction (0 on the traditional scale) to both *Pt* races (Table 1). Three of these (5-BIL42, Cannizzo, and Orfeo), as well as the accession Ethiopia, were also immune to *Pgt* races. The accession PI 572849 and accessions MG 15516/1, Nauryz 2, and Bezenchukskaya 139 were fully susceptible (4 on the traditional scale of infection type) to the *Pt* and *Pgt* races, respectively.

In this study, the broad-sense heritability (*H*^2^) value was high for resistance to all *Pt* and *Pgt* races, ranging between 89.7% and 93.6%, while the genetic advance (GA) ranged from 5.2 to 6.4 (see Table 2). The highest GA and *H*^2^ were obtained for the *Pt* race TQTGT, while the lowest values of GA and *H*^2^ were observed for the *Pgt* race THMTF.

Analysis of variance (ANOVA) of *Pt* and *Pgt* infection types revealed significant impacts of genotype, race, and the genotype × race interaction on plant resistance. The ANOVA results suggest genotype as the predominant source of resistance variation in the collection, explaining 69.5% of the total LR and 75.8% of SR resistance variance (Table 3). The second important source of resistance variation to both LR and SR was the genotype × race interaction, explaining 24.5% and 17.7% of the total variation in resistance to LR and SR, respectively. The impact of race on resistance was minimal (2.2% and 2.4% in total LR and SR resistance, respectively).

Pearson’s correlation analysis revealed a moderate positive correlation between the two *Pt* races (*r* = 0.41, *p* < 0.001) and a strong positive correlation between the two *Pgt* races (*r* = 0.59, *p* < 0.001; Table 4). There were no correlations between the *Pgt* race QHHSF and the *Pt* races, while the second *Pgt* race, THMTF, demonstrated weak positive correlations with the two *Pt* races (*p* < 0.001).

### 2.2. Adult Plant Resistance to LR and SR

**Figure 2:**
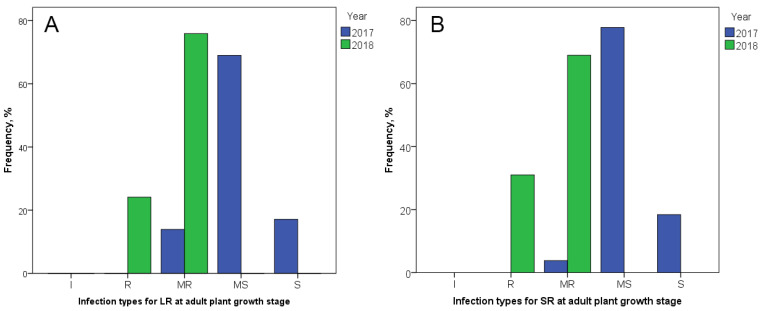
Infection types among 193 tetraploid wheat cultivars and breeding lines to (**A**) *Puccinia triticina* Eriks. (LR) and (**B**) *Puccinia graminis* f. sp. *tritici* (SR) at the adult plant stage in the field of Karabalyk agricultural experimental station in 2017 and 2018.

APR to LR and SR, assessed in the field of Karabalyk agricultural experimental station (KAES), varied significantly between two years (Figure 2). In 2017, the largest part of the studied durum collection demonstrated MS infection type in both LR (69%) and SR (77.8%). A small number of accessions showed MR (13.9% to LR and 3.8% to SR) and S (17.1% to LR and 18.4% to SR) infection types, and no I or R reactions were observed. In 2018, in contrast, the majority of the collection presented MR to LR (75.9%) and SR (69%) with a minor R infection type (24.1% to LR and 31% to SR). Infection types MS and S were not registered in 2018. Thus, the studied tetraploid wheat collection was more susceptible to LR and SR in 2017 and more resistant in 2018.

Ten accessions demonstrated the best mean level of resistance to LR (MR), and five to SR (MR) at the adult plant stage (Table 5). Four of them (Pedroso, PI 157985, Zenit, and Neodur) were MR to both LR and SR. The lowest mean level of resistance to LR (S) was observed in three accessions: PI 134946, PI 68287, and PI 286075. PI 134946, PI 68287, and PI 289606 were also susceptible to SR.

Correlation analysis revealed no significant correlations of APR to *Pt* with seedling resistance to *Pt* races, but weak positive correlations with *Pgt* races (*r* = 0.21–0.31, *p* < 0.05; Table 6). APR to *Pgt* was positively correlated with the *Pgt* race QHHSF (*r* = 0.18, *p* < 0.05) and negatively correlated with the *Pt* race TQTGT (*r* = −0.16, *p* < 0.05). Correlation between APR to *Pt* and *Pgt* in the field was also positive (*r* = 0.56, *p* < 0.01). The severity of leaf and stem rust infections was negatively correlated with two yield-related traits—the weight of kernels per spike (WKP) and grain yield per m^2^ (GY; *r* = from −0.15 to −0.33, *p* < 0.05)—but positively correlated with thousand kernel weight (TKW; *r* = 0.17–0.35, *p* < 0.05). Correlations with the number of fertile spikes (NFS) were not significant.

### 2.3. Identification of QTLs Associated with LR and SR Resistance Using GWAS

Overall, 38 QTLs were identified for LR and SR resistance, including 17 QTLs for LR resistance (Table 7) and 21 QTLs for SR resistance (Table 8). QTLs associated with resistance to LR were found on 8 of 14 tetraploid wheat chromosomes (2A, 2B, 3A, 3B, 6A, 6B, 7A, and 7B; Table 7). *p*-values for identified QTLs ranged from 1.1 × 10^−4^ to 8.8 × 10^−4^, and their effect on plant resistance was between 0.4 and 1.5 points on a 9-point scale. The phenotypic variation explained by the individual SNPs varied from 5.9 to 8.8%. Among the 17 QTLs for LR resistance, 6 QTLs were detected for APR in different years, 6 QTLs were for resistance to the race TGTGT at the seedling stage, 3 QTLs were for the race TQTGT also at the seedling stage, 1 QTL was for the race TGTGT at the seedling growth stage and APR, and 1 QTL was for both races at the seedling stage.

GWAS analysis identified 21 QTLs for SR resistance at different plant growth stages, comprising 9 QTLs for APR in different years, 6 QTLs for QHHSF, 5 QTLs for THMTF at the seedling stage, and 1 QTL for both races and APR (Table 8). All of these QTLs were detected on 11 of 14 chromosomes, except for 4A, 4B, and 7B. Their *p*-values ranged between 2.3 × 10^−5^ and 9.9 × 10^−4^, and the effect on the resistance to SR was from 0.2 to 1.8 on a 9-point scale. The phenotypic variation explained by the individual SNPs ranged between 6.2 and 9.7%.

Two SNP markers were found to be associated with both LR and SR resistance. Marker IWB60584 on chromosome 3B was associated with seedling resistance to *Pt* race TGTGT and *Pgt* race QHHSF, while SNP IWB7431 on chromosome 7A was associated with seedling resistance to *Pt* race TGTGT and APR resistance to SR (Table 7 and Table 8).

### 2.4. Comparison of QTLs with Genes for LR and SR Resistance and Resistance QTLs from Literature Survey

**Figure 3:**
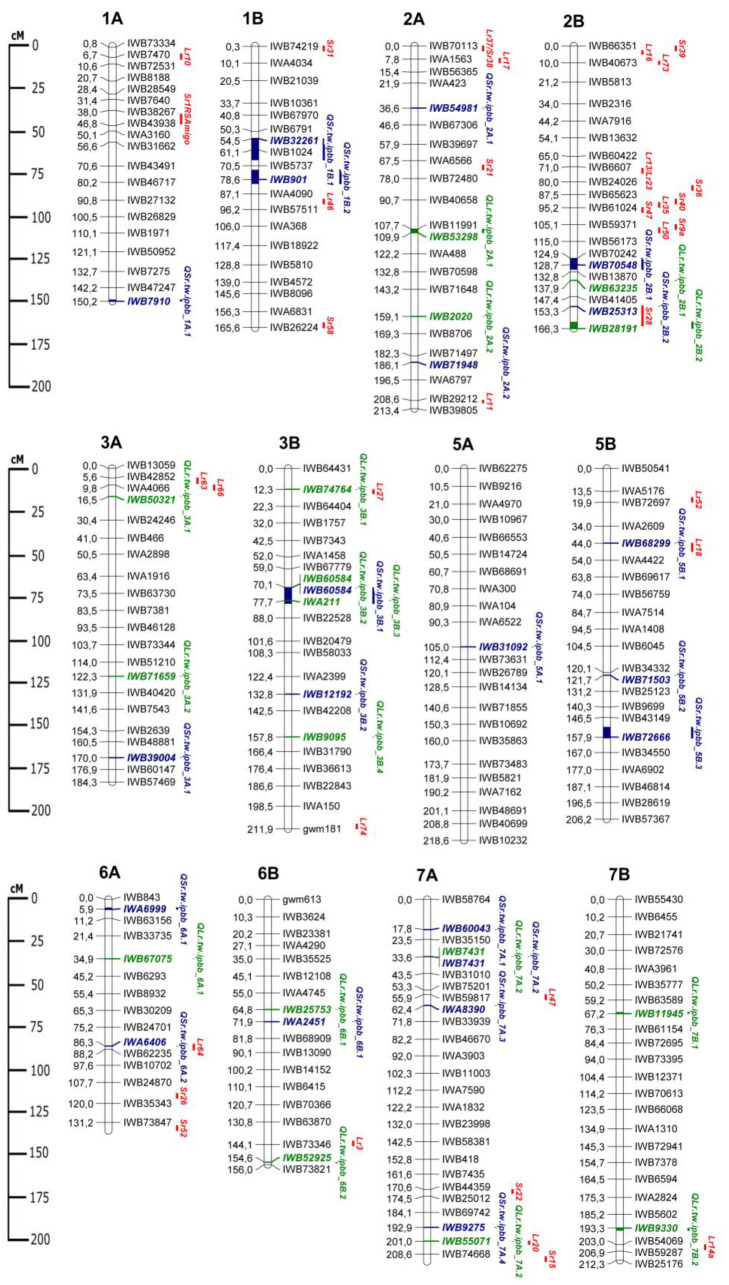
Genetic map of QTLs associated with resistance to leaf rust (LR) and stem rust (SR), as well as some possible candidate resistance genes. The SNP names are shown on the right and marker positions are shown on the left of the chromosome, in centimorgans (cM). Significant markers, the QTLs identified in this study, and potential candidate resistance genes are highlighted in color: red for genes, green for LR QTLs, and blue for SR QTLs.

The QTLs identified in this study were compared with the positions of previously reported QTLs for LR and SR resistance, as well as with *Lr* and *Sr* genes. For broader comparison, candidate rust resistance QTLs and genes of both tetraploid and hexaploid wheat were considered. The genetic map of QTLs from this study, with important *Lr* and *Sr* genes, is illustrated in Figure 3. Among *Lr* genes, possible candidates were found for *QLr.tw.ipbb_3A.1* (*Lr63* and *Lr66*), *QLr.tw.ipbb_3B.1* (*Lr27*), *QLr.tw.ipbb_6B.2* (*Lr3*), *QLr.tw.ipbb_7A.2* (*Lr20*), and *QLr.tw.ipbb_7B.2* (*Lr14a*). These QTLs and genes were positioned close to each other. A candidate *Sr* gene was found only for QTL *QSr.tw.ipbb_2B.2* (*Sr28*). QTLs for SR resistance *QSr.tw.ipbb_5B.1*, *QSr.tw.ipbb_6A.2*, and *QSr.tw.ipbb_7A.3* were located close to the LR resistance genes *Lr18*, *Lr64*, and *Lr47*, respectively.

In addition to already-known resistance genes, QTLs from the current study were compared with LR and SR resistance QTLs from other sources (Table 9).

Comparative analysis with the literature sources helped to identify possible candidate rust resistance QTLs and genes for 11 out of 17 LR resistance QTLs detected in the current study (Table 9). Two candidate QTLs have been previously described in both tetraploid and hexaploid wheat types; the other seven QTLs were exclusive for hexaploid wheat, including three *Lr* genes, and two QTLs mapped closely to genes *Lr20* and *Lr14a*. The remaining six QTLs associated with LR resistance identified in the current study had no similarities to *Lr* genes or QTLs from other sources and, as such, may be considered to be novel. A comparison of QTLs identified for SR resistance with other QTLs from previous works revealed candidates for 16 out of 21 QTLs (Table 9). Among them, six QTLs had close positions to SR resistance QTLs described in both tetraploid and hexaploid wheat, seven candidate QTLs were found in tetraploid wheat only, and the other three candidate QTLs were mapped in hexaploid wheat. The remaining five QTLs for SR resistance identified in the current study were mapped far from previously reported resistance QTLs.

## 3. Discussion

### 3.1. Resistance to LR and SR in the Studied Tetraploid Wheat Collection at Seedling and Adult Plant Growth Stages

In the current study, a diverse germplasm collection of tetraploid wheat accessions from different regions in the world was evaluated for resistance to *Pt* and *Pgt* races common in Kazakhstan. The high genetic diversity of the studied tetraploid wheat collection can be explained in terms of its composition, including seven *T. turgidum* subspecies (*T. durum*, *T. turanicum*, *T. polonicum*, *T. turgidum*, *T. carthlicum*, *T. dicoccum*, and *T. dicoccoides*). Interestingly, seven out of eight accessions with absolute resistance to both *Pt* races belonged to the subspecies *T. durum* (5-BIL42, Athena, Cannizzo, Kronos, Orfeo, Tito, and Tiziana), while the remaining accession (PI 289606) belonged to *T. polonicum* (see Table 1). Geographically, 5-BIL42, Athena, Cannizzo, Orfeo, Tito, and Tiziana originated in Italy, while Kronos was from the U.S. and PI 289606 was from the U.K. (Supplementary Table S1). The most susceptible to *Pt* accession was PI 573182 (*T. carthlicum* from Turkey). At the adult plant growth stage, the reaction of wheat to *Pt* was studied for two years, and the results varied between years. In 2017, the largest part of the studied collection demonstrated MS reaction to LR while, in 2018, MR reaction prevailed (Figure 1). The larger amount of precipitation in 2017, especially in the period May–June (Table 10), presumably provided more favorable conditions for the development of the disease, and so the severity was significantly higher. The mean values for LR resistance in the two years were used to determine the most resistant accessions. The list of accessions with the best LR resistance level (MR) included Pedroso, PI 157985, Zenit, Neodur, Primadur, Brindur, CLTR11390, PI 278350, PI 330554, and PI 352488 (Table 5). These accessions belong to different subspecies of tetraploid wheat with different countries of origin (Supplementary Table S1). Among the studied tetraploid wheat accessions, two demonstrated the lowest resistance to LR in the field (S): PI 134946 (*T. polonicum* from Portugal) and PI 68287 (*T. turanicum* from Azerbaijan) (Table 5). When considering the two wheat growth stages, no matches in resistant and susceptible accessions were found.

Assessment of seedling resistance to *Pgt* races revealed four highly resistant accessions: 5-BIL42, Cannizzo, Orfeo (*T. durum* from Italy), and the accession Ethiopia (*T. dicoccoides* with unknown origin; see Table 1). The first three of them were also resistant at the seedling stage to *Pt* races. Meanwhile, accessions MG 15516/1 (*T. dicoccum* from Syria), Nauryz 2 (*T. durum* from Kazakhstan), and Bezenchukskaya 139 (*T. durum* from Russia) were totally susceptible to *Pgt* races (Table 1). As for APR resistance to SR, its severity was also presumably influenced by the amount of precipitation in 2017 (Figure 1). The mean values of resistance over the two years were determined, demonstrating the best levels of resistance (MR) in Pedroso, PI 157985, Zenit, Neodur, and PI 223171 (Table 5). The first four of these accessions were also MR to LR. Three accessions demonstrated the highest susceptibility to SR (S) at the adult plant growth stage (Table 5). One of them (PI 289606) was mentioned above as the most resistant to *Pt* at the seedling stage, while the other two (PI 134946 and PI 68287) were the most susceptible to LR. The matches between LR- and SR-resistant accessions, as well as between LR and SR susceptible accessions (Table 5), positive correlations between APR resistances to LR and SR (Table 6), and positive correlations between *Pgt* race THMTF and two *Pt* races (Table 4), may indicate the presence of common genetic factors controlling resistance to these two rust pathogens. As *Pt* and *Pgt* are close relatives [71], loci providing multiple resistance may be involved. The pleiotropic gene clusters *Lr34/Yr18/Pm38/Sr57* [72], *Lr46/Yr29/Pm39/Sr58* [73], and *Lr67/Yr46/Pm46/Sr55* [74] have been previously described for resistance to wheat fungal diseases.

Rust epidemics among tetraploid and hexaploid wheat are quite common worldwide, including in Kazakhstan, where information on the resistance genes in local breeding lines and cultivars of tetraploid wheat is very limited [45]. Cultivars and breeding lines of tetraploid wheat highlighted as the most resistant in this study deserve special attention as sources of genetic resistance for wheat breeding programs. The seedling resistance and APR to LR and SR assessed in the current study demonstrated wide ranges and levels of variability. High heritability values, ranging from 89.7% for the race THMTF (*Pgt*) to 93.6% for the race TQTGT (*Pt*; Table 2), high GAM values (>20%; Table 2), and the impact of genotype on seedling resistance (69.5% in LR and 75.8% in SR; Table 3), together with the high genetic diversity in the studied tetraploid wheat collection, provided a promising source for GWAS analysis.

### 3.2. QTLs Identified for LR and SR Resistance and Comparison of Them to Genes and QTLs from the Literature

Previous studies on QTL mapping and GWAS for rust resistance in tetraploid and hexaploid wheat have identified a large number of QTLs and MTAs [75] (Table 9), in addition to 80 *Lr* and 60 *Sr* genes, which have already been confirmed [22]. In this study, using a collection of 193 tetraploid wheat accessions harvested in Kazakhstan, 38 QTLs both for LR and SR resistances were identified in the seedling (greenhouse) and adult (KAES, North Kazakhstan) plant growth stages (Table 7 and Table 8 and Figure 2). QTLs for LR resistance in both growth stages were compared to *Lr* genes and previously reported QTLs. The positions of two LR QTLs (*QLr.tw.ipbb_7A.2* and *QLr.tw.ipbb_7B.2*) were close to the genes *Lr20* and *Lr14a*, respectively (Figure 3 and Table 9). One QTL, *QLr.tw.ipbb_7A.2*, was identified at the seedling stage only for the *Pt* race TQTGT (Table 7). Gene *Lr20*, associated with this QTL, has been obtained from *T. aestivum* [76], providing resistance at all growth stages, but has previously been described as ineffective in Kazakhstan [11]. QTL *QLr.tw.ipbb_7B.2* was detected at both growth stages (Table 7). Gene *Lr14a* originated from *T. diccocoides*, and provides resistance at the seedling stage, but only MS level at the adult plant stage [77]. Previously, it has described been as moderately effective against LR in the northern regions of Kazakhstan [11]. Three LR QTLs (*QLr.tw.ipbb_3A.1*, *QLr.tw.ipbb_3B.1*, and *QLr.tw.ipbb_6B.2*) were positioned close to *Lr* genes (*Lr63/Lr66*, *Lr27*, and *Lr3*, respectively) and overlapped with LR resistance QTLs reported in the literature (Table 9). Genes *Lr3* and *Lr27* originated from *T. aestivum*, and provided moderate resistance at all growth stages [78,79]; *Lr63* was from *Triticum monococcum* [80]; and *Lr66* was from *Aegilops speltoides* [81]. LR resistance QTLs similar to *QLr.tw.ipbb_2A.1* and *QLr.tw.ipbb_6B.1* have been observed previously in tetraploid and hexaploid wheat (Table 9). Four other LR resistance QTLs—*QLr.tw.ipbb_2A.2*, *QLr.tw.ipbb_2B.1*, *QLr.tw.ipbb_2B.2*, and *QLr.tw.ipbb_3B.2*—were close to QTLs identified in hexaploid wheat (Table 9).

Among the QTLs identified in this study for SR resistance, only QTL *QSr.tw.ipbb_2B.2* was positioned close to the known SR resistance gene *Sr28* (Figure 3 and Table 9). *Sr28* is a gene providing high resistance to SR at all growth stages [82]. QTLs similar to *QSr.tw.ipbb_2B.2* have also been described for tetraploid and hexaploid wheat (Table 9). Seven QTLs for SR resistance were identified in the current study—*QSr.tw.ipbb_1A.1*, *QSr.tw.ipbb_1B.2*, *QSr.tw.ipbb_2A.1*, *QSr.tw.ipbb_5B.2*, *QSr.tw.ipbb_5B.3*, *QSr.tw.ipbb_6A.2*, and *QSr.tw.ipbb_7A.3*—which were in similar genetic locations to resistance QTLs from other published studies on tetraploid wheat; three similar QTLs were found in hexaploid wheat, and six similar QTLs were described for both tetraploid and hexaploid wheat (Table 9).

### 3.3. Potentially Novel LR and SR Resistance Loci for Durum Wheat

Six QTLs—*QLr.tw.ipbb_3A.2*, *QLr.tw.ipbb_3B.3*, *QLr.tw.ipbb_3B.4*, *QLr.tw.ipbb_6A.1*, *QLr.tw.ipbb_7A.1*, *QLr.tw.ipbb_7B.1*—on chromosomes 3A, 3B, 6A, 7A, and 7B were significantly associated with resistance to LR, but did not overlap with known *Lr* genes or QTLs of tetraploid and hexaploid wheat and, therefore, can be considered as novel LR resistance loci (Figure 3 and Table 9). Three of them were APR QTLs (*QLr.tw.ipbb_3A.2*, *QLr.tw.ipbb_6A.1*, *QLr.tw.ipbb_7B.1*), detected at the adult plant growth stage (Table 7). The other three (*QLr.tw.ipbb_3B.3*, *QLr.tw.ipbb_3B.4*, *QLr.tw.ipbb_7A.1*) were identified at the seedling growth stage (Table 7).

Among the SR resistance QTLs identified in this study, five loci—*QSr.tw.ipbb_2A.2*, *QSr.tw.ipbb_3A.1*, *QSr.tw.ipbb_5B.1*, *QSr.tw.ipbb_7A.1*, *QSr.tw.ipbb_7A.2*—on chromosomes 2A, 3A, 5B, and 7A were detected in chromosome regions that were non-overlapping with any known *Sr* genes or QTLs of wheat (Figure 3 and Table 9). We consider these QTLs as being novel for tetraploid wheat SR resistance. Three of these QTLs (*QSr.tw.ipbb_3A.1*, *QSr.tw.ipbb_7A.1*, and *QSr.tw.ipbb_7A.2*) were APR loci, while the remaining two (*QSr.tw.ipbb_2A.2* and *QSr.tw.ipbb_5B.1*) were identified at the seedling stage for *Pgt* race QHHSF (Table 8).

Summarizing the above, the genetic locations of the majority of resistance QTLs identified in this study were consistent with previously reported resistance genes or QTLs of tetraploid and hexaploid wheat, or were in the vicinity of known genetic resistance factors. This provides a strong indication of the reliability of the conducted GWAS. Still, 35% and 24% of the LR and SR QTLs, respectively, can be considered novel genetic factors for LR and SR resistance, requiring further research.

## 4. Materials and Methods

### 4.1. Germplasm and Genotyping

The tetraploid wheat collection assessed for seedling and adult leaf and stem rust resistance included 193 accessions of various origins [49] (Supplementary Table S1). The seeds of 191 accessions were provided by the Research Centre for Cereal and Industrial Crops (CREA; Foggia, Italy), and two accessions (Bezenchukskaya 139 and Nauryz 2) were provided by the Research Institute of Biological Safety Problems (RIBSP, South Kazakhstan) as susceptible check cultivars. Details of the genetic diversity, population structure, and linkage disequilibrium (LD) patterns of this collection of tetraploid wheat have been previously described in [83] and [84]. The genotyping data for 16,425 SNP markers (Illumina iSelect 90K wheat SNP assay; TraitGenetics GmbH, Gatersleben, Germany) were provided by Nicola Pecchioni and Giovanni Laidò (Research Centre for Cereal and Industrial Crops, Foggia, Italy). SNP markers with less than 10% missing data and with minor allele frequency (MAF) greater than 10% were retained [84].

### 4.2. Assessment of Seedling Resistance and Adult Plant Resistance to Leaf and Stem Rust

For the comprehensive study, the plant response of the 193 tetraploid wheat cultivars to *Pt* and *Pgt* pathogens was evaluated at the seedling and adult plant growth stages. Assessment of seedling resistance was performed in the greenhouse (GH) at the RIBSP under controlled conditions. For the inoculation of wheat seedlings (7–10 days after sowing) in the greenhouse, two *Pt* races (TGTGT and TQTGT) and two *Pgt* races (THMTF and QHHSF) were used [8,15]. The collection was inoculated with each race separately in two random independent replicates. These races have been common in wheat-growing regions of Kazakhstan for the last five years [15]. Inoculated plants were placed in boxes in the greenhouse with appropriate temperature conditions (22 ± 2 °C for SR, 18 ± 2 °C for LR) and illumination (10,000–15,000 lux; 16 h light period) [85,86,87]. Plant reaction was assessed on the 14th day after the inoculation of seedlings with fully expanded first leaves, according to the scale reported by Stakman [88]. Plants showing infection type 0 were considered immune (I); 1, resistant (R); 2, moderately resistant (MR); 3, moderately susceptible (MS); and 4, susceptible (S).

Disease screening at the adult stage of plant development in the field was conducted at Karabalyk Agricultural Experimental Station (KAES) in the Kostanay region, North Kazakhstan, in the 2017 and 2018 growing seasons. Table 10 summarizes the details of the meteorological conditions during the vegetation period (May–August) for these two years.

Field experiments were conducted according to a randomized complete block design with two independent replicates. Each accession was planted in two rows, at 25 seeds per row, with a row spacing of 15 cm, and kept under rainfed conditions. In KAES17 and KAES18, inoculation with local *Pt* and *Pgt* pathogens occurred under uncontrolled natural conditions. The disease severity of the wheat plants was evaluated using the modified Cobb scale [89], and host response to infection was evaluated as described in [6]. The assessment was performed at the stage of grain ripening with the maximum level of disease manifestation. To meet the data format required for GWAS, the results of seedling resistance and APR were converted to the 0–9 linear disease scale [90] (Supplementary Table S2). Thus, the analysis was conducted for four independent environments: race-specific seedling resistance under greenhouse (controlled) conditions (1), non-race-specific adult plant resistance (APR) at KAES fields (uncontrolled conditions) in 2017 (2) and 2018 (3), and mean values of 2017 and 2018 (4).

To determine the influence of plant resistance on yield-related traits, the tetraploid wheat panel was evaluated for the number of fertile spikes (NFS, pcs), number of kernels per spike (NKS, pcs), number of kernels per plant (NKP, pcs), the weight of kernels per plant (WKP, g), thousand-kernel weight (TKW, g), and grain yield per m^2^ (GY, g/m^2^).

### 4.3. Statistical and Association Mapping Analysis

Pearson’s correlation, ANOVA, and other descriptive statistics were analyzed using the R software METAN package [91]. Broad-sense heritability [92], and genetic advance in absolute units (GA) and as a percentage of the mean (GAM) were estimated, in accordance with the methods described by Johnson et al. [93], using the Variability package in R.

GWAS was performed using the GAPIT R software package (v3) [94]. Marker–trait associations (MTAs) between SNP markers and agronomic traits were detected using a mixed linear model (MLM) [95] with the kinship matrix (K) and the population structure matrix (Q) determined previously through Bayesian methods using STRUCTURE software [96], with the optimum number of sub-populations (*K*) being equal to three [56]. The map distance at which the LD fell below the r^2^ threshold of 0.3 was used to define the confidence intervals for QTLs detected in this study, as previously reported in the literature [97]. The threshold for significant associations was *p* < 0.001. The strength of the models was visualized using a cumulative quantile–quantile (QQ) plot of expected vs. observed *p*-values. For convenience, hereinafter, MTAs will be denoted as QTLs. Neighboring MTAs linked to each other were merged into one common QTL.

The consensus map of tetraploid wheat described in [98] was used to assign the genomic location of SNP markers of identified QTLs. The genetic map, with identified QTLs and some important *Lr* and *Sr* genes, was constructed using MapChart 2.2 software (Wageningen University & Research, Netherlands) [99]. The genetic position of the identified QTLs was compared with data obtained in other studies on tetraploid and hexaploid wheat resistance to LR and SR. The sequences of the SNP-tagged markers within the estimated interval of each QTL were used as queries in a BLAST search against the durum wheat genome on the InterOmics Svevo portal website (https://d-data.interomics.eu, accessed on 23 March 2022). The output of this search was the hit match corresponding to markers with physical positions. These positions were compared with QTLs associated with the traits considered in the present study, using the Genome Annotation Viewer (http://d-gbrowse.interomics.eu, accessed on 25 March 2022) on the InterOmics website (www.interomics.eu, accessed on 25 March 2022).

## 5. Conclusions

The study of a tetraploid wheat germplasm panel demonstrated diverse reaction types to LR and SR pathogens at both seedling and adult plant growth stages. The high genetic diversity of the panel provided a good basis for the identification of loci associated with resistance to LR and SR. As a result of the GWAS (*p* < 0.001), 38 QTLs—including 17 for LR and 21 for SR resistance—were identified. Among the LR resistance QTLs, 10 were detected at the seedling stage, 6 were APR QTLs, and 1 QTL was for both seedling and adult resistances. The genetic positions of 11 LR resistance QTLs coincided with the positions of *Lr* genes and QTLs, while the remaining 6 were presumably novel resistant factors. For SR resistance, there were 11 QTLs at the seedling stage, 9 QTLs at the adult plant stage, and 1 QTL at both the seedling and adult stages. Among them, five SR resistance QTLs were newly discovered. A high level of resistance (0) at the seedling stage to two *Pt* and two *Pgt* races was observed in five (Athena, Kronos, Tito, Tiziana, and PI 289606) and one (Ethiopia) wheat accessions, respectively, while three accessions (5-BIL42, Cannizzo, and Orfeo) were highly resistant (0) to both pathogens. Six wheat accessions demonstrated the best level of resistance (MR) to LR at the adult plant growth stage (Primadur, Brindur, CLTR11390, PI 278350, PI 330554, and PI 352488). Accession PI 223171 had the MR reaction type to SR at the adult growth stage, while four accessions (Pedroso, PI 157985, Zenit, and Neodur) were MR to both LR and SR. The QTLs and resistant wheat accessions identified in this work can potentially be used for MAS of tetraploid and hexaploid wheat and for the breeding of new, highly productive LR- and SR-resistant cultivars.

## Figures and Tables

**Table 1:** Descriptive statistics of *Pt* and *Pgt* infection types at the seedling stage.

Pathogen (Races)	Mean IT *	Immune Genotypes (IT)	Susceptible Genotypes (IT)
*Pt* (TGTGT and TQTGT)	2+	5-BIL42, Athena, Cannizzo, Kronos, Orfeo, Tito, Tiziana, PI 289606 (0)	PI 573182 (4)
*Pgt* (QHHSF and THMTF)	2+	5-BIL42, Cannizzo, Orfeo, Ethiopia (0)	MG 15516/1, Nauryz 2, Bezenchukskaya 139 (4)

*—mean values for the studied collection, IT—infection type.

**Table 2:** Statistical parameters for the reaction of the tetraploid wheat collection to races of *P. triticina* Eriks. (LR) and *Puccinia* *graminis* f. sp. *tritici* (SR) at the seedling stage.

Disease	Race	CV	*H*^2^ (%)	GA	GAM (%)
LR	TGTGT	23.3	90.1	5.2	137.7
	TQTGT	19.4	93.6	6.4	148.0
SR	QHHSF	23.2	93.4	5.7	173.4
	THMTF	21.6	89.7	5.2	124.5

LR—leaf rust, SR—stem rust, CV—coefficient of variation, *H*^2^—broad-sense heritability, GA—genetic advance, GAM—genetic advance as a percentage of the mean.

**Table 3:** Two-way ANOVA of infection type of leaf rust (LR) and stem rust (SR) at the seedling stage.

Disease	Factor	df	SS	MS	F-value	*p*-Value	% SS
LR	Genotype	192	5129.5	26.7	36.3	3.4 × 10^−171^	69.5
	Race	1	52.9	52.9	71.7	5.3 × 10^−16^	2.2
	Genotype × Race	192	1804.1	9.4	12.8	1.6 × 10^−95^	24.5
	Residuals	384	282.9	0.7			3.8
SR	Genotype	192	5020	26.1	37.2	3 × 10^−173^	75.8
	Race	1	148.9	148.9	212.1	1.5 × 10^−38^	2.4
	Genotype × Race	192	1171.4	6.1	8.7	3.6 × 10^−71^	17.7
	Residuals	384	269.5	0.7			4.1

LR—leaf rust, SR—stem rust, df—degree of freedom, SS—the sum of squares, MS—mean squares.

**Table 4:** Correlations among seedling resistance to *Pt* races TGTGT and TQTGT and *Pgt* races QHHSF and THMTF.

	*Pt*_TGTGT	*Pt*_TQTGT	*Pgt*_QHHSF	*Pgt*_THMTF
*Pt*_TGTGT	1	0.41 ***	0.10 ^ns^	0.30 ***
*Pt*_TQTGT	0.41 ***	1	0.11 ^ns^	0.34 ***
*Pgt*_QHHSF	0.10 ^ns^	0.11 ^ns^	1	0.59 ***
*Pgt*_THMTF	0.30 ***	0.34 ***	0.59 ***	1

***—*p* < 0.001, ^ns^—not significant.

**Table 5:** Descriptive statistics of the mean APR to LR and SR evaluated at Karabalyk agricultural experimental station in 2017 and 2018.

Infection	Min *	Max *	Mean *	Resistant Genotypes (IT)	Susceptible Genotypes (IT)
LR	MR	S	MR	Pedroso, PI 157985, Zenit, Neodur, Primadur, Brindur, CLTR11390, PI 278350, PI 330554, PI 352488 (MR)	PI 134946, PI 68287, PI 286075 (S)
SR	MR	S	MR	Pedroso, PI 157985, Zenit, Neodur, PI 223171 (MR)	PI 289606, PI 134946, PI 68287 (S)

*—minimal, maximal, and mean values for the studied collection, LR—leaf rust, SR—stem rust, MR—moderately resistant, S—susceptible, IT—infection type.

**Table 6:** Correlations among adult plant resistance to *Pt* and *Pgt* and yield components in the field of Karabalyk agricultural experimental station and seedling resistance under greenhouse conditions.

	*Pt*_APR	*Pt*_TGTGT	*Pt*_TQTGT	*Pgt*_QHHSF	*Pgt*_THMTF	NFS	WKP	TKW	GY
*Pt*_APR	1	−0.05 ^ns^	−0.09 ^ns^	0.31 *	0.21 *	0 ^ns^	−0.2 *	0.17 *	−0.33 *
*Pgt*_APR	0.56 **	−0.13 ^ns^	−0.16 *	0.18 *	0.08 ^ns^	−0.09 ^ns^	−0.15 *	0.35 *	−0.3 *

*—*p* < 0.05, **—*p* < 0.01, ^ns^—not significant, APR—adult plant resistance, NFS—number of fertile spikes, WKP—weight of kernels per spike, TKW—thousand kernel weight, GY—grain yield per m^2^.

**Table 7:** QTLs for resistance to leaf rust (LR) identified in tetraploid wheat.

#	QTL	SNP	Chr.	Position (cM)	*p*-Value	R2 (%)	Allele	Effect	Environment
1	*QLr.tw.ipbb_2A.1*	IWB53298	2A	107.7–109.9	1.7 × 10^−4^	7.6	G	1.2	GH (TGTGT)
2	*QLr.tw.ipbb_2A.2*	IWB2020	2A	159.1	7.9 × 10^−4^	6.3	A	1.1	GH (TQTGT)
3	*QLr.tw.ipbb_2B.1*	IWB63235	2B	137.9–138.1	6.1 × 10^−4^	6.2	A	1.0	GH (TQTGT)
4	*QLr.tw.ipbb_2B.2*	IWB28191	2B	166.3	4.0 × 10^−4^	6.4	T	0.4	APR_KAES17, APR_KAES_mean
5	*QLr.tw.ipbb_3A.1*	IWB50321	3A	16.5	3.8 × 10^−4^	6.7	C	1.2	GH (TGTGT)
6	*QLr.tw.ipbb_3A.2*	IWB71659	3A	122.3	8.5 × 10^−5^	8.8	G	0.5	APR_KAES17, APR_KAES_mean
7	*QLr.tw.ipbb_3B.1*	IWB74764	3B	12.3	7.6 × 10^−4^	6.4	A	0.5	APR_KAES17
8	*QLr.tw.ipbb_3B.2*	IWB60584	3B	70.1	8.4 × 10^−4^	5.9	A	1.5	GH (TGTGT)
9	*QLr.tw.ipbb_3B.3*	IWA211	3B	77.7	3.9 × 10^−4^	6.8	A	1.4	GH (TQTGT, TGTGT)
10	*QLr.tw.ipbb_3B.4*	IWB9095	3B	157.8	7.9 × 10^−4^	6.0	C	1.2	GH (TGTGT)
11	*QLr.tw.ipbb_6A.1*	IWB67075	6A	34.9	5.2 × 10^−4^	6.8	G	0.6	APR_KAES_mean
12	*QLr.tw.ipbb_6B.1*	IWB25753	6B	64.8	6.4 × 10^−4^	6.2	A	1.0	GH (TGTGT)
13	*QLr.tw.ipbb_6B.2*	IWB52925	6B	154.6–155.1	4.0 × 10^−4^	7.1	A	0.5	APR_KAES17, APR_KAES_mean
14	*QLr.tw.ipbb_7A.1*	IWB7431	7A	33.6	8.8 × 10^−4^	5.9	A	0.9	GH (TGTGT)
15	*QLr.tw.ipbb_7A.2*	IWB55071	7A	201	4.9 × 10^−4^	6.4	A	1.0	GH (TQTGT)
16	*QLr.tw.ipbb_7B.1*	IWB11945	7B	66.2–67.2	7.6 × 10^−4^	6.4	T	0.5	APR_KAES17
17	*QLr.tw.ipbb_7B.2*	IWB9330	7B	193.3–194.8	1.1 × 10^−4^	8.1	C	1.0	GH (TGTGT), APR_KAES17

QTL—quantitative trait locus (loci), SNP—single nucleotide polymorphism, Chr.—chromosome, R2—phenotypic variation explained by the QTL; GH—greenhouse; APR—adult plant resistance; KAES—Karabalyk agricultural experimental station.

**Table 8:** QTLs for resistance to stem rust (SR) identified in tetraploid wheat.

#	QTL	SNP	Chr.	Position (cM)	*p*-Value	R2 (%)	Allele	Effect	Environment
1	*QSr.tw.ipbb_1A.1*	IWB7910	1A	150.2	1.9 × 10^−4^	7.2	G	1.0	GH (QHHSF)
2	*QSr.tw.ipbb_1B.1*	IWB32261	1B	54.5–67.2	4.2 × 10^−4^	7.2	T	0.7	APR_KAES18, APR_KAES_mean
3	*QSr.tw.ipbb_1B.2*	IWB901	1B	72.9–81.2	8.7 × 10^−4^	6.4	G	0.4	GH (THMTF, QHHSF), APR_KAES_mean
4	*QSr.tw.ipbb_2A.1*	IWB54981	2A	36.6	9.9 × 10^−4^	6.2	T	0.7	APR_KAES_mean
5	*QSr.tw.ipbb_2A.2*	IWB71948	2A	186.1	2.6 × 10^−4^	6.9	G	0.8	GH (QHHSF)
6	*QSr.tw.ipbb_2B.1*	IWB70548	2B	124.9–131.6	4.2 × 10^−4^	7.2	A	0.5	APR_KAES17
7	*QSr.tw.ipbb_2B.2*	IWB25313	2B	153.3	8.0 × 10^−4^	6.0	A	0.9	GH (THMTF)
8	*QSr.tw.ipbb_3A.1*	IWB39004	3A	170	7.5 × 10^−4^	6.5	A	0.6	APR_KAES_mean
9	*QSr.tw.ipbb_3B.1*	IWB60584	3B	70.1–79.6	8.6 × 10^−4^	7.2	A	1.5	GH (QHHSF)
10	*QSr.tw.ipbb_3B.2*	IWB12192	3B	132.8	7.5 × 10^−4^	7.7	C	0.2	APR_KAES18
11	*QSr.tw.ipbb_5A.1*	IWB31092	5A	105	4.1 × 10^−4^	6.4	G	1.6	GH (QHHSF)
12	*QSr.tw.ipbb_5B.1*	IWB68299	5B	44	7.0 × 10^−4^	5.9	T	1.1	GH (QHHSF)
13	*QSr.tw.ipbb_5B.2*	IWB71503	5B	121.7	5.0 × 10^−4^	6.5	C	0.8	GH (THMTF)
14	*QSr.tw.ipbb_5B.3*	IWB72666	5B	151.9–158.6	1.5 × 10^−4^	8.4	C	0.5	APR_KAES_mean
15	*QSr.tw.ipbb_6A.1*	IWA6999	6A	4.8-5.9	2.3 × 10^−5^	9.7	G	1.8	GH (THMTF)
16	*QSr.tw.ipbb_6A.2*	IWA6406	6A	86.3	1.4 × 10^−4^	7.8	G	1.6	GH (THMTF)
17	*QSr.tw.ipbb_6B.1*	IWA2451	6B	71.9	3.9 × 10^−4^	7.3	C	0.6	APR_KAES17
18	*QSr.tw.ipbb_7A.1*	IWB60043	7A	17.8	5.1 × 10^−4^	6.9	G	0.5	APR_KAES_mean
19	*QSr.tw.ipbb_7A.2*	IWB7431	7A	33.6	2.2 × 10^−4^	7.9	G	0.4	APR_KAES17, APR_KAES_mean
20	*QSr.tw.ipbb_7A.3*	IWA8390	7A	62.4	6.4 × 10^−4^	6.2	T	0.8	GH (THMTF)
21	*QSr.tw.ipbb_7A.4*	IWB9275	7A	192.9–193	3.9 × 10^−4^	6.5	A	1.0	GH (QHHSF)

QTL—quantitative trait locus (loci), SNP—single nucleotide polymorphism, Chr.—chromosome, R2—phenotypic variation explained by the QTL; GH—greenhouse; APR—adult plant resistance; KAES—Karabalyk agricultural experimental station.

**Table 9:** Comparison of leaf rust (LR) and stem rust (SR) resistance QTLs with possible candidate QTLs from the literature.

Leaf Rust (LR)					
#	QTL	Position (cM)	Candidate Leaf Rust QTLs		Candidate *Lr* Genes
			Tetraploid Wheat	Hexaploid Wheat	
1	*QLr.tw.ipbb_2A.1*	107.7–109.9	[43]	[50]	
2	*QLr.tw.ipbb_2A.2*	159.1		[51]	
3	*QLr.tw.ipbb_2B.1*	137.9–138.1		[30]	
4	*QLr.tw.ipbb_2B.2*	166.3		[30]	
5	*QLr.tw.ipbb_3A.1*	16.5		[30]	*Lr63*, *Lr66*
6	*QLr.tw.ipbb_3A.2*	122.3			
7	*QLr.tw.ipbb_3B.1*	12.3		[50]	*Lr27*
8	*QLr.tw.ipbb_3B.2*	70.1		[51]	
9	*QLr.tw.ipbb_3B.3*	77.7			
10	*QLr.tw.ipbb_3B.4*	157.8			
11	*QLr.tw.ipbb_6A.1*	34.9			
12	*QLr.tw.ipbb_6B.1*	64.8	[52]	[53]	
13	*QLr.tw.ipbb_6B.2*	154.6–155.1		[54]	*Lr3*
14	*QLr.tw.ipbb_7A.1*	33.6			
15	*QLr.tw.ipbb_7A.2*	201			*Lr20*
16	*QLr.tw.ipbb_7B.1*	66.2–67.2			
17	*QLr.tw.ipbb_7B.2*	193.3–194.8			*Lr14a*
**Stem Rust (SR)**					
**#**	**QTL**	**Position (cM)**	**Candidate Stem Rust QTLs**		**Candidate *Sr* Genes**
			**Tetraploid Wheat**	**Hexaploid Wheat**	
1	*QSr.tw.ipbb_1A.1*	150.2	[55]		
2	*QSr.tw.ipbb_1B.1*	54.5–67.2	[56]	[23,31]	
3	*QSr.tw.ipbb_1B.2*	72.9–81.2	[57]		
4	*QSr.tw.ipbb_2A.1*	36.6	[57]		
5	*QSr.tw.ipbb_2A.2*	186.1			
6	*QSr.tw.ipbb_2B.1*	124.9–131.6	[55,58]	[59]	
7	*QSr.tw.ipbb_2B.2*	153.3	[57,58]	[60]	*Sr28*
8	*QSr.tw.ipbb_3A.1*	170			
9	*QSr.tw.ipbb_3B.1*	70.1–79.6	[57]	[61]	
10	*QSr.tw.ipbb_3B.2*	132.8		[62]	
11	*QSr.tw.ipbb_5A.1*	105	[57]	[63]	
12	*QSr.tw.ipbb_5B.1*	44			
13	*QSr.tw.ipbb_5B.2*	121.7	[64,65]		
14	*QSr.tw.ipbb_5B.3*	151.9–158.6	[57]		
15	*QSr.tw.ipbb_6A.1*	4.8–5.9		[66,67,68]	
16	*QSr.tw.ipbb_6A.2*	86.3	[57]		
17	*QSr.tw.ipbb_6B.1*	71.9	[69]	[23]	
18	*QSr.tw.ipbb_7A.1*	17.8			
19	*QSr.tw.ipbb_7A.2*	33.6			
20	*QSr.tw.ipbb_7A.3*	62.4	[58,69]		
21	*QSr.tw.ipbb_7A.4*	192.9–193		[67,70]	

**Table 10:** Rainfall and temperature during vegetation periods in 2017 and 2018 at Karabalyk Agricultural Experimental Station.

Environment	Average Temperature, °C					Rainfall, mm				
	May	June	July	August	Mean	May	June	July	August	Overall
KAES17	13.5	18.7	19.7	20.3	18.1	50.9	79.3	69.7	37.8	237.7
KAES18	12.1	17.0	22.8	18.3	17.6	32.7	46.5	78.7	39.6	197.5

KAES17—Karabalyk Agricultural Experimental Station 2017; KAES18—Karabalyk Agricultural Experimental Station 2018.

## Data Availability

All data is available as supplementary files.

## Supplementary Materials

The following supporting information can be downloaded at: https://www.mdpi.com/article/10.3390/plants11151904/s1, Table S1. The list of 193 tetraploid wheat accessions involved in the study. Table S2. Leaf rust and stem rust resistance in studied tetraploid wheat collection at seedling and adult plant growth stages.
